# Comparison and trend of perioperative outcomes between robot-assisted radical prostatectomy and open radical prostatectomy: nationwide inpatient sample 2009-2014

**DOI:** 10.1590/S1677-5538.IBJU.2019.0420

**Published:** 2020-07-31

**Authors:** Yingyi Qin, Hedong Han, Yongping Xue, Cheng Wu, Xin Wei, Yuzhou Liu, Yang Cao, Yiming Ruan, Jia He

**Affiliations:** 1 Second Military Medical University Department of Health Statistics Shanghai China Department of Health Statistics, Second Military Medical University, Shanghai, China; 2 Second Military Medical University Changhai Hospital Department of Urology Shanghai China Department of Urology, Changhai Hospital, Second Military Medical University, Shanghai, China; 3 Mount Sinai St. Luke's and West Medical Center New York USA Mount Sinai St. Luke's and West Medical Center, New York, USA; 4 Virginia Commonwealth University Department of Cardiology Richmond USA Department of Cardiology, Virginia Commonwealth University, Richmond, USA; 5 Karolinska Institutet Institute of Environmental Medicine Unit of Biostatistics Stockholm Sweden Unit of Biostatistics, Institute of Environmental Medicine, Karolinska Institutet, Stockholm, Sweden; 6 Örebro University School of Medical Sciences Clinical Epidemiology and Biostatistics Örebro Sweden Clinical Epidemiology and Biostatistics, School of Medical Sciences, Örebro University, Örebro, Sweden; 7 Tongji University School of Medicine Shanghai China Tongji University School of Medicine, Shanghai, China

**Keywords:** Prostatic Neoplasms, Prostatectomy, Robotics

## Abstract

**Purpose:**

To make a further evaluation of perioperative outcomes between the robot-assisted radical prostatectomy (RARP) and open radical prostatectomy (ORP), we conducted a comparison and trend analysis by using the Nationwide Inpatient Sample (NIS) from 2009 to 2014.

**Materials and Methods:**

Adult prostate cancer patients with radical prostatectomy were abstracted from the NIS. RARP and ORP were identified according to the International Classification of Diseases, 9^th^ Revision, Clinical Modification procedure codes. The perioperative outcomes included blood transfusion, intraoperative and postoperative complications, prolonged length of stay (pLOS), and in-hospital mortality. Propensity score matching method and multivariable logistic regression model were performed to adjust for the pre-defined covariates. The annual percent change (APC) was used to detect the change trend of rates for outcomes.

**Results:**

A total of 77.054 patients were included in our study. According to the results of propensity score matching analyses, RARP outperformed ORP in blood transfusion (1.96% vs. 9.40%), intraoperative complication (0.73% vs. 1.25%), overall postoperative complications (8.87% vs. 11.97%), and pLOS (13.39% vs. 36.70%). We also found that there was a significant decreasing tendency of incidence in blood transfusion (APC=-9.81), intraoperative complication (APC=-12.84), and miscellaneous surgical complications (APC=-14.09) for the RARP group. The results of multivariable analyses were almost consistent with those of propensity score matching analyses.

**Conclusions:**

The RARP approach has lower incidence rates of perioperative complications than the ORP approach, and there is a potential decreasing tendency of complication incidence rates for the RARP.

## INTRODUCTION

Prostate cancer (PCa) is one of the most common solid organ cancer in men, and accounts for about 20% of all cancers diagnosed (1). For localized PCa, the surgery with radical prostatectomy (RP) is the dominant approach (2). RP could be performed by open or minimally invasive approach. According to the Prostate Cancer Guidelines provided by European Association of Urology (EAU), there is no recommendation for the surgical approach choice among open, laparoscopic and robot-assisted approaches (3). The robot-assisted radical prostatectomy (RARP), which was first reported by Binder and Kramer in 2001, is becoming the dominant surgical approach for RP (4). In the United States, more than 60 percent of RPs had been performed through robot-assisted approach in 2009 (5).

About the pros and cons of RARP, several studies had found that RARP was associated with reductions in some intraoperative and postoperative complications when compared with open radical prostatectomy (ORP) (5–7). RARP was also reported to reduce the possibility of blood transfusions and shorten the length of stay in hospital. However, some researchers thought that RARP and ORP had comparable rates of operative complications (8–10). A randomized controlled trail (RCT) had shown that minimally invasive benefits were seen in the RARP group and both approaches yielded similar functional outcomes at 12 weeks and 2 years (11, 12). These previous studies had provided some useful but conflicting information about the comparison between these two surgical approaches. Further evidence should be based on the larger population study, and the trend of comparison results along with the development and wide application of RARP need to be investigated. The aim of our study was to make further evaluation of comparison of perioperative complication rates between RARP and ORP and investigate the trend of perioperative complication rates for these two approaches using six years of Nationwide Inpatient Sample (NIS) data (2009-2014).

## MATERIALS AND METHODS

### Data source

The database used in this study was based on inpatient discharge data obtained from the NIS of the Healthcare Cost and Utilization Project (HCUP). A robot-assisted modifier code was introduced and received approval by the US Food and Drug Administration (FDA) to identify robot-assisted procedures in October 1, 2008. Therefore, we chose sample population from all of NIS inpatient discharge data from January 2009 to December 2014. Detailed information on NIS data is available at <http://www.hcup-us.ahrq.gov>. The NIS is a deidentified database so institutional review board approval is not required.

### Patients and surgical approach

Adult patients (older than 18 years) with a primary diagnosis of PCa were identified using the International Classification of Diseases, 9^th^ Revision, Clinical Modification (ICD-9-CM) codes 185.0. The surgical approaches were classed according to the ICD-9-CM procedure codes. All PCa patients with radical prostatectomy were selected (ICD-9-CM 60.5). The RARP group was defined as PCa patients who underwent the robotic assisted procedures (ICD-9-CM 17.4×). The ORP group was defined as patients who underwent RA without the robotic assisted procedures and laparoscopy procedures (ICD-9-CM 54.21).

### Demographic characteristics

The following demographic characteristics of each patient were abstracted from the database: age, year of surgery (2009-2014), race, Elixhauser Comorbidity Index (ECI), insurance status. The ECI includes 29 disease conditions and might be a useful way to control for confounding in cancer outcomes research (13, 14). Hospital-related characteristics were also included in our study: location, academic status, region, control/ownership of hospital, bed size of hospital.

### Outcomes

Intraoperative complications: The intraoperative complications were identified according to ICD-9-CM code 998.2 suggested by previous study (5), and included accidental puncture or laceration during surgery.

Postoperative complications: We included seven categories of complications (Cardiac, Respiratory, Genitourinary, Wound, Vascular, Miscellaneous medical, and Miscellaneous surgical) using ICD-9-CM code provided by Hu et al. (7, 15).

Homologous blood transfusion: We identified patients with blood transfusion using ICD- 9-CM procedure codes 99.02 and 99.04.

Prolonged length of stay (pLOS): The pLOS was defined as length of stay beyond the 75^th^ percentile (2 days).

In-hospital mortality: The information of in-hospital mortality was abstracted from “DIED” variable.

### Statistical analysis

Descriptive statistics were summarized for the demographic characteristics of the RARP group and the ORP group. Median (interquartile range, IQR) was calculated for age, and counts (percentages) were calculated for other categorical variables. We conducted the comparison of demographic characteristics between two groups by using Wilcoxon Rank Sum test, Chi-square test, or Cochran-Mantel-Haenszel (CMH) test.

We conducted the propensity score matching method to balance the covariates between the RARP group and the ORP group. The logistic regression model was performed to calculate the propensity score based on all covariates described in above demographic characteristics. The RARP and ORP group were matched based on the logit of the propensity score by using the calipers of width equal to 0.2 of the standard deviation of the logit of the propensity score (16). The odds ratios (OR) and corresponding 95% confidence interval (CI) of RARP compared with ORP for each perioperative outcome were calculated by using univariate logistic regression in matched cohort and using multivariable logistic regression model adjusted for all demographic characteristics in non-matched cohort.

We used the annual percent change (APC) to investigate the trend of rate change for blood transfusion, pLOS, intraoperative and postoperative complication, from 2009 to 2014, respectively (17). Similar to the overall analyses shown above, we also calculated the ORs of each year for these perioperative outcomes. To detect the change trend of ORs, we constructed the annual ratio change (ARC) based on the theory of APC calculation. For example, if the ARC is 1.01, and the OR is 1.05 in 2009, the OR is 1.05×1.01 = 1.06 in 2010. The APC over 0 and the ARC over 1 represent a trend of increase, and on the contrary represent a trend of decrease.

All statistical analyses were performed using SAS software (version 9.4; SAS Institute Inc., Cary, NC), and the statistical figures were drawn by using R software. All reported p values were two-sided and a p value <0.05 was regarded as statistically significant.

## RESULTS

### Demographic characteristics

**Figure 1:**
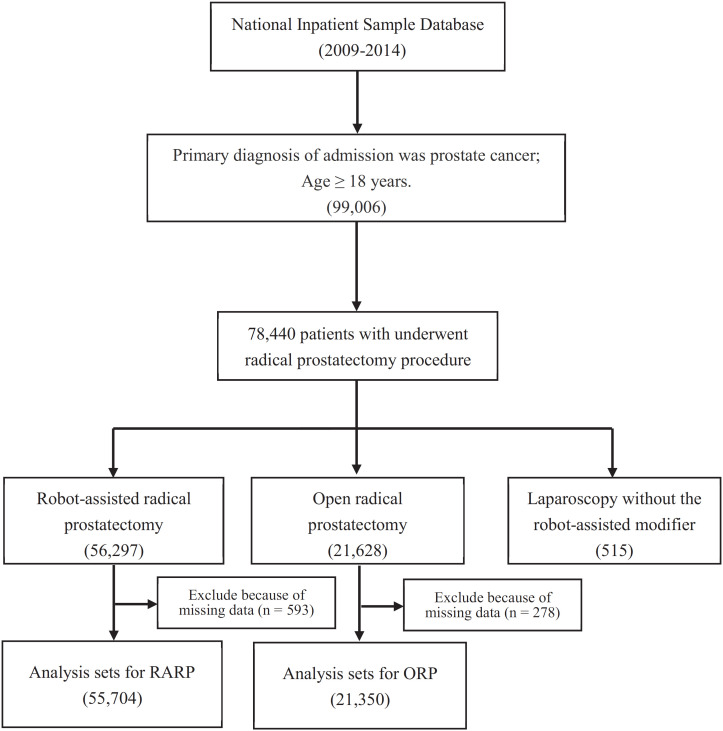
The flow chart of patients selection.

Among a total of 99.006 adult patients with primary diagnosis of PCa between 2009 and 2014, 78,440 patients underwent radical prostatectomy procedure. There were 55.704 patients with RARP approach and 21.350 patients with ORP approach (Figure-1).

According to the distribution of demographic characteristics, we found that the number of patients with ORP approach had reduced from 2009 to 2014. The proportion of RARP on white was higher than that of ORP (68.69% vs 66.24%), and more black patients underwent ORP approach (10.58% vs. 12.48%). Greater proportion of the RARP group had no comorbidities than the ORP group (for ECI of 0: 34.51% vs. 32.41%). With regard to the hospital-related characteristics, we found that RARP approach would be more likely to be performed in urban (97.89% vs. 91.19%, p <0.0001) and teaching (72.67% vs. 59.75%, p <0.0001) hospitals than ORP approach. There was statistical significant difference on all of the characteristics between the RARP group and the ORP group, and the baseline covariates were much more balanced after conducting the propensity score matching procedure. Further details of demographic characteristics before and after matching are listed in Table-1.

**Table 1:** Demographic characteristics of patients treated with radical prostatectomy for prostate cancer.

		Pre-propensity score-matched			Post-propensity score-matched		
		RARP (N=55704)	ORP (N=21350)	p value	RARP (N=20573)	ORP (N=20573)	p value
Age, year, median (IQR)		62 (57-67)	62 (57-67)	0.0086	62 (57-67)	62 (57-67)	0.9041
**Year of surgery, n (%)**							
	2009	9608 (17.25)	5450 (25.53)	<0.0001	5089 (24.74)	5154 (25.05)	0.2171
	2010	8819 (15.83)	4410 (20.66)		4064 (19.75)	4190 (20.37)	
	2011	11338 (20.35)	4858 (22.75)		4713 (22.91)	4683 (22.76)	
	2012	8978 (16.12)	2556 (11.97)		2510 (12.20)	2503 (12.17)	
	2013	8661 (15.55)	2192 (10.27)		2193 (10.66)	2171 (10.55)	
	2014	8300 (14.90)	1884 (8.82)		2004 (9.74)	1872 (9.10)	
**Race, n (%)**							
	white	38261 (68.69)	14142 (66.24)	<0.0001	13699 (66.59)	13650 (66.35)	0.6484
	black	5894 (10.58)	2665 (12.48)		2560 (12.44)	2543 (12.36)	
	Hispanic	3099 (5.56)	1207 (5.65)		1098 (5.34)	1176 (5.72)	
	Asian or Pacific Islander	914 (1.64)	273 (1.28)		251 (1.22)	273 (1.33)	
	Native American	281 (0.50)	72 (0.34)		67 (0.33)	68 (0.33)	
	other	1915 (3.44)	533 (2.50)		540 (2.62)	519 (2.52)	
	missing	5340 (9.59)	2458 (11.51)		2358 (11.46)	2344 (11.39)	
**ECI, n (%)**							
	0	19226 (34.51)	6920 (32.41)	<0.0001	6704 (32.59)	6711 (32.62)	0.9440
	1	20383 (36.59)	7584 (35.52)		7357 (35.76)	7345 (35.70)	
	2	10795 (19.38)	4391 (20.57)		4216 (20.49)	4233 (20.58)	
	>3	5300 (9.51)	2455 (11.50)		2296 (11.16)	2284 (11.10)	
**Insurance status, n (%)**							
	Medicare	18452 (33.13)	6923 (32.43)	<0.0001	6567 (31.92)	6643 (32.29)	0.8146
	Medicaid	1110 (1.99)	695 (3.26)		655 (3.18)	634 (3.08)	
	Private insurance	34084 (61.19)	12549 (58.78)		12219 (59.39)	12179 (59.20)	
	Other	2058 (3.69)	1183 (5.54)		1132 (5.50)	1117 (5.43)	
**Hospital location, n (%)**							
	Rural	1175 (2.11)	1881 (8.81)	<0.0001	1168 (5.68)	1179 (5.73)	0.8151
	Urban	54529 (97.89)	19469 (91.19)		19405 (94.32)	19394 (94.27)	
**Hospital academic status, n (%)**							
	Nonteaching	15224 (27.33)	8594 (40.25)	<0.0001	7872 (38.26)	7835 (38.08)	0.7073
	Teaching	40480 (72.67)	12756 (59.75)		12701 (61.74)	12738 (61.92)	
**Hospital region, n (%)**							
	Northeast	10554 (18.95)	4020 (18.83)	<0.0001	3930 (19.10)	3885 (18.88)	0.7744
	Midwest	13407 (24.07)	5337 (25.00)		5090 (24.74)	5109 (24.83)	
	South	19380 (34.79)	7893 (36.97)		7619 (37.03)	7572 (36.81)	
	West	12363 (22.19)	4100 (19.20)		3934 (19.12)	4007 (19.48)	
**Control/ownership of hospital, n (%)**							
	Government, nonfederal	5289 (9.49)	2154 (10.09)	<0.0001	2074 (10.08)	2023 (9.83)	0.6162
	Private, non-profit	45613 (81.88)	16855 (78.95)		16254 (79.01)	16331 (79.38)	
	Private, invest-own	4802 (8.62)	2341 (10.96)		2245 (10.91)	2219 (10.79)	
**Bed size of hospital, n (%)**							
	Small	7682 (13.79)	2266 (10.61)	<0.0001	2219 (10.79)	2232 (10.85)	0.1593
	Medium	12244 (21.98)	4622 (21.65)		4354 (21.16)	4522 (21.98)	
	Large	35778 (64.23)	14462 (67.74)		14000 (68.05)	13819 (67.17)	t

**RARP**=Robot-assisted Radical Prostatectomy; **ORP**=Open Radical Prostatectomy; **IQR**=Interquartile Range; **ECI**=Elixhauser Comorbidity Index.

### Perioperative outcomes

The information of comparison for perioperative outcomes between the RARP group and the ORP group are shown in Table-2. The results of pre-propensity score-matched cohort and post-propensity score-matched cohort were consistent. According to the results of propensity score matching analyses, we found that the RARP group had significant lower rates of almost all of the pre-defined complications and outcomes than the ORP group except vascular complication (0.40% vs. 0.52%, OR=0.77, 95% CI: 0.58-1.03, p=0.0802). The results indicated that patients with RARP had lower incidence rate for blood transfusion (1.96% vs. 9.40%, p <0.0001), intraoperative complication (0.73% vs. 1.25%, p <0.0001), and overall postoperative complications (8.87% vs. 11.97%, p <0.0001). Additionally, the RARP group also had shorter LOS (for pLOS, 13.39% vs. 36.70%, p <0.0001).

**Table 2:** Perioperative outcomes during hospitalization of patients treated with RARP and ORP.

		Pre-propensity score-matched				Post-propensity score-matched			
		RARP (N=55704)	ORP (N=21350)	RARP vs. ORP OR (95% CI)*	p value	RARP (N=20573)	ORP (N=20573)	ORP OR (95% CI)#	RARP vs. p value
Homologous blood transfusion		896 (1.61)	2032 (9.52)	0.18 (0.16-0.19)	<0.0001	403 (1.96)	1934 (9.40)	0.19 (0.17-0.21)	<0.0001
Intraoperative complication		385 (0.69)	266 (1.25)	0.62 (0.52-0.73)	<0.0001	151 (0.73)	257 (1.25)	0.58 (0.48-0.72)	<0.0001
**Postoperative complication**									
	Overall	4502 (8.08)	2576 (12.07)	0.71 (0.68-0.75)	<0.0001	1825 (8.87)	2463 (11.97)	0.72 (0.67-0.76)	<0.0001
	Cardiac	513 (0.92)	292 (1.37)	0.75 (0.65-0.88)	0.0003	203 (0.99)	281 (1.37)	0.72 (0.60-0.86)	0.0004
	Respiratory	640 (1.15)	508 (2.38)	0.57 (0.50-0.64)	<0.0001	271 (1.32)	477 (2.32)	0.56 (0.48-0.65)	<0.0001
	Genitourinary	491 (0.88)	259 (1.21)	0.78 (0.67-0.92)	0.0023	190 (0.92)	247 (1.20)	0.77 (0.63-0.93)	0.0063
	Wound	198 (0.36)	133 (0.62)	0.66 (0.52-0.83)	0.0004	84 (0.41)	130 (0.63)	0.64 (0.49-0.85)	0.0018
	Vascular	210 (0.38)	110 (0.52)	0.82 (0.64-1.04)	0.1052	82 (0.40)	106 (0.52)	0.77 (0.58-1.03)	0.0802
	Miscellaneous medical	2678 (4.81)	1400 (6.56)	0.80 (0.74-0.85)	<0.0001	1070 (5.20)	1338 (6.50)	0.79 (0.73-0.86)	<0.0001
	Miscellaneous surgical	917 (1.65)	591 (2.77)	0.67 (0.60-0.74)	<0.0001	395 (1.92)	567 (2.76)	0.69 (0.61-0.79)	<0.0001
Length of stay >2d		6610 (11.87)	7956 (37.26)	0.25 (0.24-0.26)	<0.0001	2755 (13.39)	7551 (36.70)	0.27 (0.25-0.28)	<0.0001
In-hospital mortality		11 (0.02)	14 (0.07)	0.28 (0.12-0.63)	0.0021	3 (0.01)	14 (0.07)	0.21 (0.06-0.75)	0.0154

**RARP**=Robot-assisted Radical Prostatectomy; **ORP=**Open Radical Prostatectomy; **OR=**Odds Ratio; **CI=**Confidence interval.

Note: * The ORs and corresponding 95% CI for perioperative outcomes were estimated by using multivariable Logistic regression adjusted for demographic characteristics.

# The ORs and corresponding 95% CI for perioperative outcomes were estimated by using univariate Logistic regression.

### The trends of perioperative outcomes from 2009 to 2014

**Figure 2:**
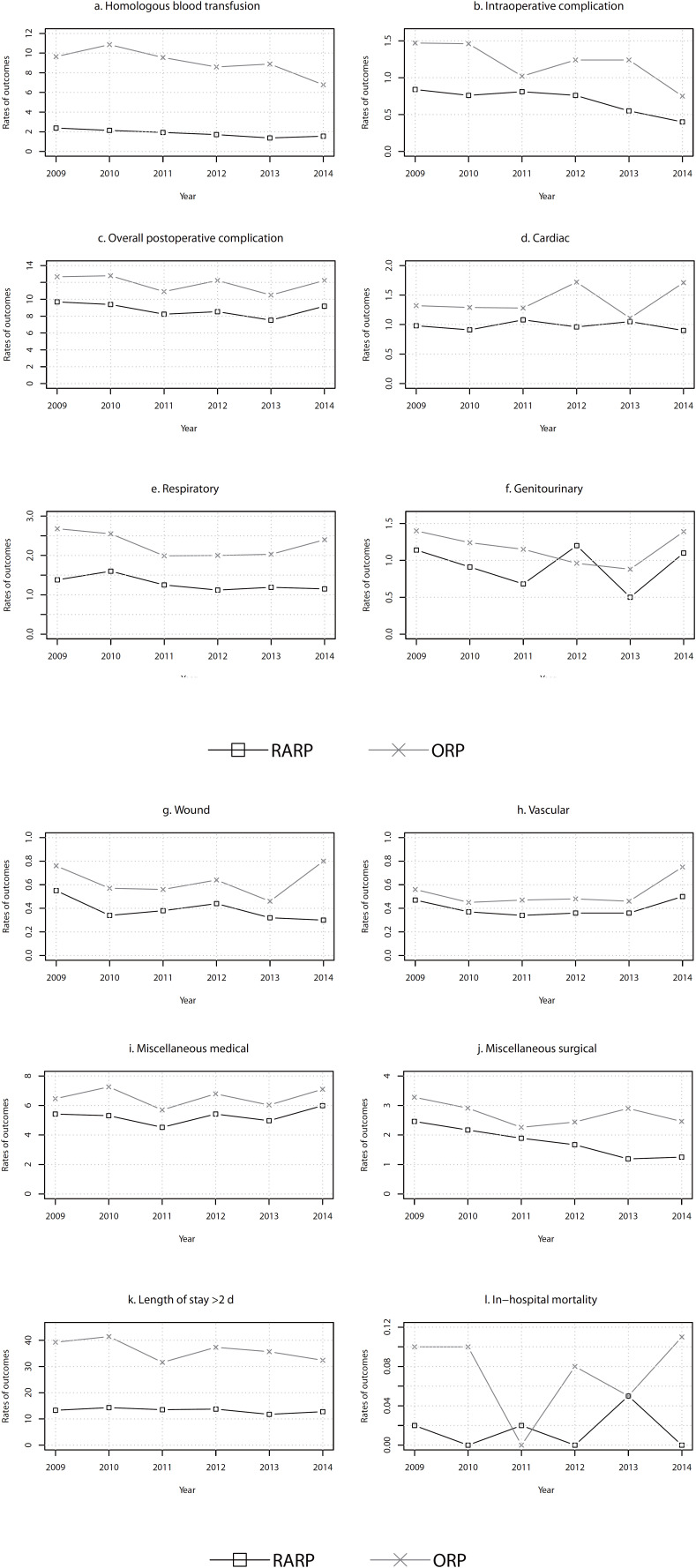
The incidence rates of the perioperative outcomes for the Robot-assisted Radical Prostatectomy group and the Open Radical Prostatectomy group from 2009 to 2014 (post-propensity score-matched cohort).

**Supplementary Figure-1:**
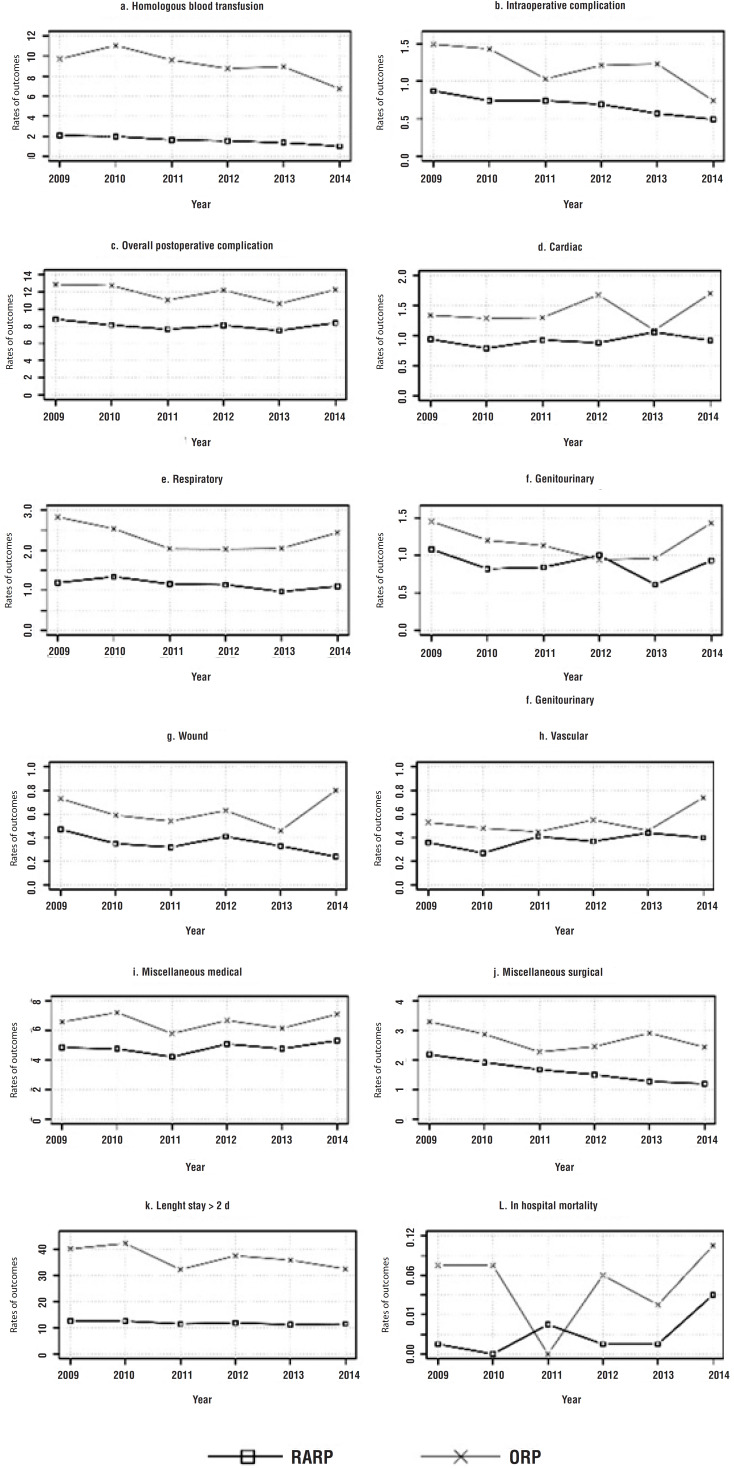
The incidence rates of the perioperative outcomes for the Robot-assisted Radical Prostatectomy group and the Open Radical Prostatectomy group from 2009 to 2014 (pre-propensity score-matched cohort).

We provided the rate of pre-defined outcomes for each year from 2009 to 2014 in Figure-2 (post-propensity score-matched cohort) and supplementary Figure-1 (pre-propensity score--matched cohort). The results of post-propensity score-matched cohort showed that the rates of these outcomes in the RARP group were consistent lower than the ORP group among most years. The results of trend analyses indicated that there were significant decreases in homologous blood transfusion (APC=-9.81, p=0.0060), intraoperative complication (APC=-12.84, p=0.0214), and miscellaneous surgical (APC=-14.09, p=0.0015) for the RARP group, and only in homologous blood transfusion (APC=-6.80, p=0.0404) for the ORP group. The significant trend of decrease was also found in pLOS (APC=-2.19, p=0.0422) for the RARP group within the pre-propensity score-matched cohort (Table-3).

**Table 3:** The trend of change for the perioperative outcomes from 2009 to 2014.

		RARP		ORP		ARC (95% CI)	p value
		APC (95% CI)	p value	APC (95% CI)	p value		
**Pre-propensity score-matched**							
	Homologous blood transfusion	-13.07 (-17.43, -8.49)	0.0016	-7.06 (-13.26, -0.42)	0.0421	0.94 (0.89, 0.98)	0.0224
	Intraoperative complication	-10.08 (-13.64, -6.37)	0.0019	-10.15 (-20.00, 0.91)	0.0627	1.01 (0.91, 1.14)	0.7461
**Postoperative complication**							
	Overall	-1.20 (-5.16, 2.92)	0.4580	-1.94 (-6.82.3.21)	0.3482	1.00 (0.98, 1.03)	0.6953
	Cardiac	2.02 (-4.37, 8.84)	0.4391	2.75 (-8.92, 15.92)	0.5659	0.99 (0.81, 1.20)	0.8482
	Respiratory	-3.86 (-9.05, 1.63)	0.1204	-3.84 (-12.01, 5.09)	0.2878	1.00 (0.91, 1.09)	0.9657
	Genitourinary	-4.08 (-16.37, 10.02)	0.4468	-2.61 (-14.89, 11.45)	0.6154	1.01 (0.88, 1.16)	0.8780
	Wound	-8.76 (-18.46, 2.10)	0.0864	-0.59 (-14.54, 15.65)	0.9193	0.90 (0.77, 1.06)	0.1571
	Vascular	5.13 (-5.24, 16.63)	0.2522	5.07 (-6.82, 18.48)	0.3164	1.00 (0.83, 1.20)	0.9706
Miscellaneous medical		1.88 (-3.28, 7.31)	0.3767	0.12 (-5.93, 6.57)	0.9590	1.00 (0.98, 1.03)	0.7788
Miscellaneous surgical		-11.57 (-12.94, -10.17)	<0.0001	-3.90 (-11.95, 4.88)	0.2747	0.92 (0.85, 1.00)	0.0525
	Length of stay >2d	-2.19 (-4.21, -0.13)	0.0422	-3.91 (-9.45, 1.98)	0.1362	1.03 (0.99,1.07)	0.1136
**Post-propensity score-matched**							
	Homologous blood transfusion	-9.81 (-14.54, -4.81)	0.0060	-6.80 (-12.70, -0.50)	0.0404	0.97 (0.86, 1.10)	0.5673
	Intraoperative complication	-12.84 (-21.44, -3.29)	0.0214	-9.97 (-20.23, 1.60)	0.0734	0.97 (0.85, 1.11)	0.5499
**Postoperative complication**							
	Overall	-2.52 (-8.24, 3.55)	0.3062	-1.85 (-7.11,3.71)	0.4002	0.99 (0.97, 1.02)	0.5198
	Cardiac	-0.42 (-5.77, 5.23)	0.8422	3.28 (-8.66, 16.77)	0.5066	0.96 (0.82, 1.14)	0.5783
	Respiratory	-5.33 (-11.34, 1.07)	0.0808	-3.44 (-11.55, 5.40)	0.3292	0.98 (0.90,1.06)	0.4981
	Genitourinary	-3.95 (-25.16, 23.28)	0.6772	-3.54 (-15.68, 10.34)	0.4981	1.00 (0.81, 1.23)	0.9671
	Wound	-8.56 (-18.25, 2.27)	0.0907	-0.65 (-14.74, 15.77)	0.9121	0.92 (0.79, 1.06)	0.1819
	Vascular	0.87 (-10.42, 13.58)	0.8500	4.35 (-8.51, 19.01)	0.4194	0.97 (0.93, 1.01)	0.0866
Miscellaneous medical		1.37 (-5.28, 8.48)	0.6076	0.28 (-6.49, 7.54)	0.9177	1.01 (0.97, 1.05)	0.4743
	Miscellaneous surgical	-14.09 (-18.66, -9.26)	0.0015	-3.86 (-12.04, 5.08)	0.2861	0.89 (0.78, 1.02)	0.0775
	Length of stay >2d	-2.19 (-6.04, 1.81)	0.1999	-3.50 (-9.28, 2.65)	0.1850	1.03 (0.93, 1.14)	0.4681

**RARP**=Robot-assisted Radical Prostatectomy; **ORP**=Open Radical Prostatectomy; **APC**=Annual Percent Change; **ARC**=Annual Ratio Change; **CI**=Confidence interval

**Figure 3:**
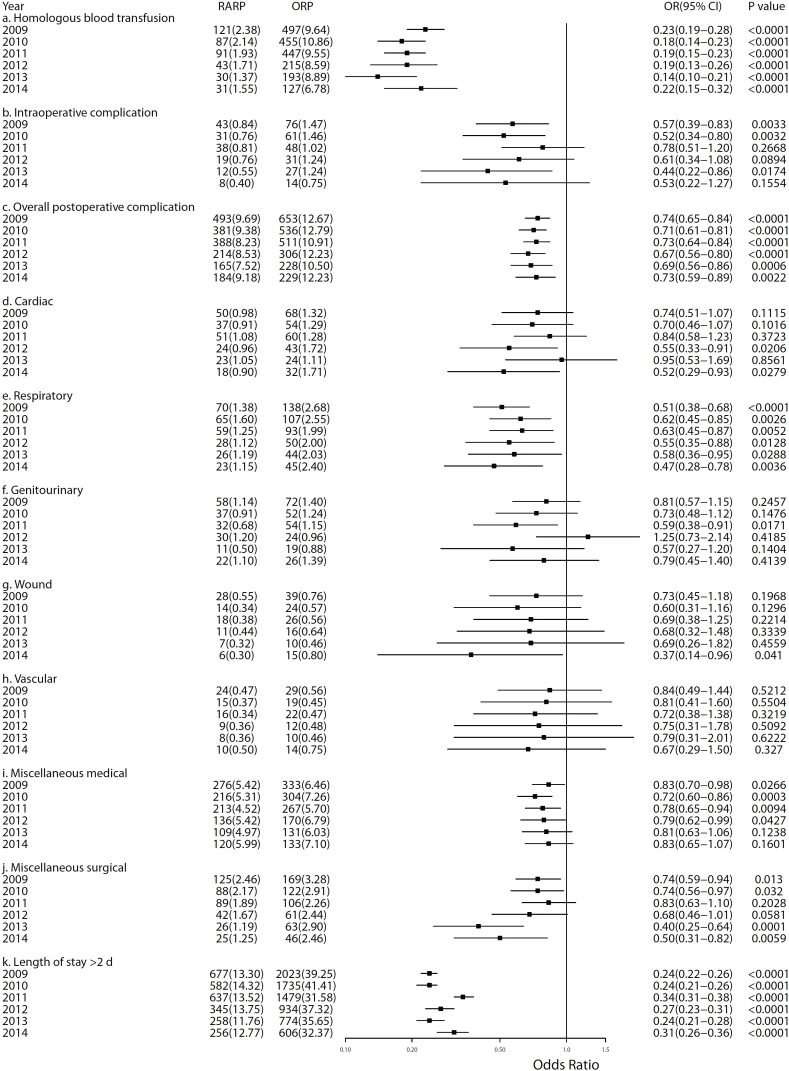
The forest plot of odds ratios for the comparison between Robot-assisted Radical Prostatectomy group with the Open Radical Prostatectomy group from 2009 to 2014 (post-propensity score-matched cohort).

**Supplementary Figure 2:**
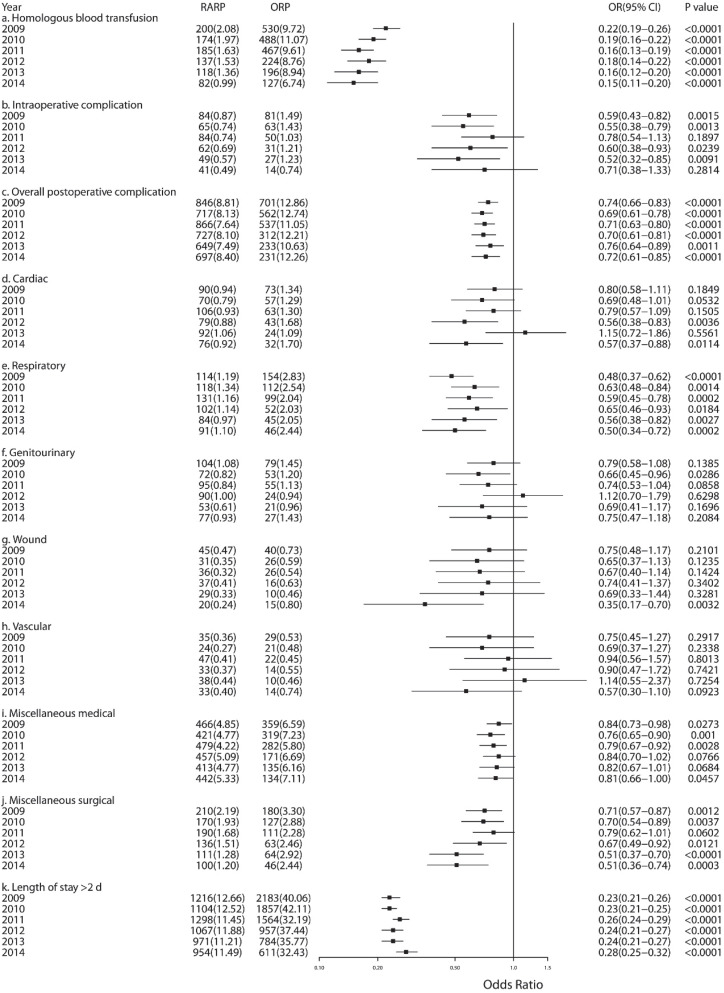
The forest plot of odds ratios for the comparison between Robot-assisted Radical Prostatectomy group with the Open Radical Prostatectomy group from 2009 to 2014 (pre-propensity score-matched cohort).

Figure-3 presents the forest plots to show the ORs (RARP vs. ORP) and corresponding 95% CI of outcomes in each year for post-propensity score-matched cohort (supplementary Figure-2 for pre-propensity score-matched cohort). The most of the OR point estimates was smaller than one for the outcomes within each year, and this indicated that the PCa patients with RARP approach would have lower risk to these complications and outcomes than patients with ORP approach. We also found the persistent significant lower risk for the RARP group in blood transfusion, overall postoperative complications, respiratory, and pLOS compared with the ORP group. According to the results of trend analyses for ORs before and after propensity score matching, there might be a potential trend of decrease in miscellaneous surgical (before matching, ARC=0.92, 95% CI: 0.851.00, p=0.0525; after matching, ARC=0.89, 95% CI: 0.78-1.02, p=0.0775, Table-3).

## DISCUSSION

The NIS database showed that the proportion of the RARP approach for PCa patients was increasing year by year in the United States, from about 60% in 2009 to about 80% in 2014. To provide further evidence for the comparison between the RARP and ORP approaches, we compared the incidence rate of pre-defined complications and outcomes between two groups by using the inpatient data form 2009 to 2014. The results indicated that the RARP group had lower rates in most of complications except vascular complication. Additionally, we also found that there were significant decreasing trend of incidence rates in blood transfusion and some surgical injuries for the RARP group, such as intraoperative complications and miscellaneous surgical complications.

According to several previous studies, they had provided reliable evidence to indicate that the RARP approach outperformed the ORP approach in blood loss and hospital stay (5, 7). The results of our study were consistent with previous finding and we further found these two superiorities in each year. With regard to the intraoperative and postoperative complications, Trinh et al. had shown that superior outcomes were seen in the RARP group, especially for intraoperative complication, cardiac, and respiratory (5), and Hu et al. had also demonstrated that fewer miscellaneous surgical complications were found in men undergoing minimally invasive RP (7). In our study, the comparison of perioperative complications between two groups showed that the RARP group had lower rates of all pre-defined complications and the significant statistical differences were detected except in vascular complication. Actually, we should notice that the incidence rates for most of these perioperative complications might be not much high in both of two surgical approaches and the absolute difference between two groups was small (e.g. only 0.22% for wound complication). The reason of large sample size for overall analyses (the information from a total of 6 years) should be taken into account for the significant statistical difference between two groups. Additionally, the results of comparison within each year had also provided the evidence to suggest the superiority of RARP approach in perioperative complications.

To explore the tendency for the rates of complications from 2009 to 2014, we calculated the APC for the RARP group and the ORP group, respectively. We found that most of APCs were less than zero for both the RARP group and the ORP group, and this had indicated a potential trend of decrease about the perioperative complications for these two surgical approaches. According to the statistical test for APC, there was significant statistical decrease tendency in blood transfusion, intraoperative complication and miscellaneous surgical for the RARP group. However, we only found significant statistical decrease tendency in blood transfusion for the ORP group. With respect to the three outcomes in the RARP group shown above, they could reflect that the injury related to the operative procedures was decreasing with the popularity of the RARP approach. However, the methods for reducing the rates of some potentially life-threatening complications, such as cardiac, respiratory, and vascular events, might be the future research direction.

Among post-propensity score-matched cohort, the results of ARC had shown that the point estimates for most of outcomes were less than 1. The small ARCs indicated that the ORs of the RARP group compared with the ORP group might be becoming smaller as the year went on, and it might also suggest that the superiority of the RARP approach become slightly obvious. Abboud et al. had suggested that the learning curve for RARP (about 100 cases) was shorter than for ORP (ranged from 250 to 1000 cases) to reduce complication rates (18). This might be a potential reason to explain the increase of advantage for RARP with the popularity of this approach.

This study was based on the inpatient information and several limitations should be considered. First, the absence of follow-up information would be a drawback to make the comprehensive evaluation for the RARP. We focused on comparing the rates and exploring the change tendency of the perioperative complications between the RARP and ORP. Several previous studies had provided some information about functional outcome, such as urinary function and sexual function (11, 12, 19, 20). Additionally, the information of mortality in our study only represented the incidence of death during the period of hospitalization. Second, we did not conduct the comparison of the cost, and there were two reasons for the lack of analyses for cost: a, we could not abstract the accurate spending which was directly related to the surgical approach; b, the adjustment of actual value for dollars within each year from 2009 to 2014 might be another challenge (21). Third, although we conducted propensity score matching method to control the bias of the acquired covariates, several unobserved confounding factors, such as stage and pathologic characteristics of cancer, the characteristics of surgeon, and the determinants of patients, should be taken into account as sources of potential bias in our study.

## CONCLUSIONS

In conclusion, our study had provided further evidence to indicate the superiority of the RARP approach in the perioperative outcomes compared to the ORP approach. The results of trend analyses indicated that there was a decreasing tendency of incidence rates for most of perioperative complications among patients underwent the RARP approach, especially for some surgical injuries related to the operative procedures. Furthermore, we concluded that the strategy for reducing potential life-threatening complications should be further investigated.

## ABBREVIATIONS

APC: annual percent change
ARC: annual ratio change
C: confidence interval
CMH: Cochran-Mantel-Haenszel
EAU: European Association of Urology
ECI: Elixhauser Comorbidity Index
FDA: Food and Drug Administration
HCUP: Healthcare Cost and Utilization Project
ICD-9-CM: International Classification of Diseases 9^th^ Revision, Clinical Modification
IQR: interquartile range
NIS: Nationwide Inpatient Sample
OR: odds ratios
ORP: open radical prostatectomy
pLOS: Prolonged length of stay
RARP: robot-assisted radical prostatectomy
RCT: randomized controlled trail
RP: radical prostatectomy
PCa: Prostate cancer.
